# Adult Women’s Blood Mercury Concentrations Vary Regionally in the United States: Association with Patterns of Fish Consumption (NHANES 1999–2004)

**DOI:** 10.1289/ehp.11674

**Published:** 2008-08-25

**Authors:** Kathryn R. Mahaffey, Robert P. Clickner, Rebecca A. Jeffries

**Affiliations:** 1 Office of Science Coordination and Policy, Office of Prevention, Pesticides, and Toxic Substances, U.S. Environmental Protection Agency, Washington, DC, USA; 2 Westat, Rockville, Maryland, USA

**Keywords:** blood, coastal, fish, mercury, NHANES, regional

## Abstract

**Background:**

The current, continuous National Health and Nutrition Examination Survey (NHANES) has included blood mercury (BHg) and fish/shellfish consumption since it began in 1999. NHANES 1999–2004 data form the basis for these analyses.

**Objectives:**

This study was designed to determine BHg distributions within U.S. Census regions and within coastal and noncoastal areas among women of childbearing age, their association with patterns of fish consumption, and changes from 1999 through 2004.

**Methods:**

We performed univariate and bivariate analyses to determine the distribution of BHg and fish consumption in the population and to investigate differences by geography, race/ethnicity, and income. We used multivariate analysis (regression) to determine the strongest predictors of BHg among geography, demographic factors, and fish consumption.

**Results:**

Elevated BHg occurred more commonly among women of childbearing age living in coastal areas of the United States (approximately one in six women). Regionally, exposures differ across the United States: Northeast > South and West > Midwest. Asian women and women with higher income ate more fish and had higher BHg. Time-trend analyses identified reduced BHg and reduced intake of Hg in the upper percentiles without an overall reduction of fish consumption.

**Conclusions:**

BHg is associated with income, ethnicity, residence (census region and coastal proximity). From 1999 through 2004, BHg decreased without a concomitant decrease in fish consumption. Data are consistent with a shift over this time period in fish species in women’s diets.

Risk of mercury-associated adverse health effects (e.g., neuropsychological deficits) in children after *in utero* methylmercury (MeHg) exposures increases as Hg exposure rises [Mergler et al. 2007; National Research Council (NRC) 2000]. Blood Hg (BHg) and hair Hg concentrations are indicators of the magnitude of MeHg exposure. National estimates for the United States are based on data from the National Health and Nutrition Examination Survey (NHANES) (Mahaffey et al. 2004; McDowell et al. 2005). Mercury concentrations in blood obtained from women of childbearing age who participated in NHANES have been collected by the National Center for Health Statistics (NCHS) on a continuing basis since 1999. Hair Hg concentrations were determined only in the years 1999 and 2000 (McDowell et al. 2005).

Although the national estimates provide an overview for the United States, indications of regional differences within the United States are suggested from local data (Bellanger et al. 2000; Karouna-Renier et al. 2008; Knobeloch et al. 2005; McKelvey et al. 2007; Ortiz-Roque and López-Rivera 2004; Sato et al. 2006; Sechena et al. 2003; Stern et al. 2001). Women residing in New York City (McKelvey et al. 2007), Hawaii (Sato et al. 2006), and Florida (Karouna-Renier et al. 2008) had both higher fish consumption and higher Hg exposure than the national estimates from NHANES (Mahaffey et al. 2004). These U.S. data, as well as international data especially from island communities (Dewailly and Pereg 2004; Hsu et al. 2007; Sakamoto et al. 2007; Soong et al. 1991; see also Bermuda Biological Stations for Research 2004) suggested the importance of regional differences and proximity to coastal areas as predictors of increased MeHg exposure. In the present article, we used the NHANES data to identify differences in distribution of BHg across the four major U.S. Census regions (Northeast, Midwest, South, and West) and between coastal and noncoastal areas of the United States.

Selecting a BHg concentration to use as an index of excessive Hg exposure depends on dose–response analysis. Over the past decade, risk assessments describing association between human exposures to MeHg and occurrence of neurologic deficits have been developed by multiple countries and organizations [Agency for Toxic Substances and Disease Research 1999; Joint Food and Agriculture Organization/World Health Organization 2003; NRC 2000; U.S. Environmental Protection Agency (EPA) 2001; European Union 2002]. We discuss these risk assessments in more detail in the Supplemental Material [see especially Table 1 (http://www.ehponline.org/members/2008/11674/suppl.pdf)].

The current reference dose (RfD) used by the U.S. EPA was based on cord blood measurements and is associated with fetal BHg concentrations of 5.8 μg/L (NRC 2000; U.S. EPA 2001). However, differences have been found between maternal and cord blood concentrations due to bioconcentration of MeHg across the placenta (Butler Walker et al. 2004; Mahaffey et al. 2004; Mergler et al. 2007; Morrissette et al. 2004; NRC 2006; Stern and Smith 2003). As a result, maternal BHg concentrations as low as approximately 3.5 μg/L may be a concern. Therefore, we refer to BHg concentrations exceeding 3.5 and 5.8 μg/L as levels of concern for the purposes of this article.

Previous analyses of NHANES BHg data (Mahaffey et al. 2004) confirmed fish and shellfish consumption as the major source of blood organic Hg for the U.S. population. In the lower portion of the range of BHg concentrations currently considered relevant to the prevention of fetal neurotoxicity (i.e., ~ 3.5 to 5.8 μg/L), organic Hg (i.e., MeHg) accounted for approximately 90% of total BHg for the general population not exposed occupationally to Hg (Mahaffey et al. 2004).

NHANES data have been used to describe the distribution of BHg concentrations in a nationally representative sample for adult women (Mahaffey et al. 2004) but have not previously been used to characterize regional and coastal exposure patterns. Analyses of NHANES data can be used to characterize major U.S. Census regions (Northeast, South, Midwest, and West) and estimate population distributions in these broad regions. Unbiased population estimates cannot be made for other U.S. geographic subdivisions, such as coastal and noncoastal regions, because weights have not been developed for them; however, distributional estimates can be made.

Explaining patterns of MeHg exposure is challenging because MeHg concentrations within and among fish species are known to vary by more than 10-fold (Mahaffey 2004; NRC 2006). Regional distributions of BHg and Hg exposure estimated from the patterns of fish consumption (i.e., Northeast, Midwest, South, and West, as well as overall coastal–noncoastal differences) remain questions.

The purposes of the present study of Hg exposures are to provide for the first time separate estimates of BHg distribution and fish consumption for the individual U.S. Census regions and for coastal and noncoastal populations. Such data are useful in determining where to focus efforts to reduce Hg exposure. The present analysis can be used to determine *a*) distributions of BHg concentrations and fish consumption that differ across the four U.S. Census regions and between the coastal and noncoastal populations, *b*) significant temporal trends in BHg levels and fish consumption between 1999 through 2004, and *c*) BHg concentrations associated with racial/ethnic and income level. Trends in patterns of fish consumption, if present, could be used to assess the impact of national fish advisories and Hg reduction programs. Data on geographic/ethnic groups and socioeconomic associations could be used to target intervention programs. The regional results can be generalized to provide population estimates for the U.S. Census regions.

## Materials and Methods

### Methodology for data analysis

We evaluated data for examinees who participated in NHANES during survey years 1999–2004 to assess the statistical association between seafood consumption and BHg by region of residence, race/ethnicity, and annual income. Further, we examined time trends for both BHg and fish consumption. NHANES is an annual survey conducted by the NCHS. The data include BHg levels, 24-hr dietary recall, and 30-day finfish and shellfish consumption frequency for women 16–49 years of age who reside in the United States. The NHANES sampling frame includes all 50 states. The documentation and publicly available data for NHANES can be found online [Centers for Disease Control and Prevention (CDC) 2006b]. The regional data are not publicly available but can be accessed by special request to the National Center for Health Statistics (NCHS) through its Research Data Center. Procedures for submitting a proposal in order to access data that are not publicly available can be found online (CDC 2006c). We performed all analyses using SAS, version 9.1 (SAS Institute Inc., Cary, NC).

Following NHANES analytic guidelines (CDC 2006a), we used SAS procedures that accurately incorporate the stratification and multistage sampling of NHANES: Proc SurveyMeans, Proc SurveyReg, and Proc SurveyFreq (SAS Institute Inc.). The weights provided by NCHS compensate for the oversampling of various subpopulations and adjust for nonresponse bias. We used weights for estimating statistics for coastal and non-coastal regions to retain these adjustments. Because we considered the variables of interest (BHg and fish consumption) to be related to some of the factors that were oversampled, we retained these adjustments to minimize the bias in the estimates. For example, NHANES over-sampled Mexican Americans, who also have lower BHg than do other racial/ethnic groups. If the weights were not used to estimate the distribution of BHg, the results would be biased low. We recognize that some bias may remain within the estimates because the weights were not specifically created for the geographic regions of coastal and noncoastal; however, these subdivisions (e.g., coastal, noncoastal, Pacific, Atlantic) are based on counties, the primary sampling units of NHANES (CDC 2006a). All multivariate analyses were done unweighted because factors associated with the dependent variables and for which over-sampling was based were included as covariates and thus adjusted for in the modeling.

In order to estimate long-term Hg intake, we combined data collected through the 24-hr dietary recall and the 30-day fish frequency questionnaire to estimate 30-day Hg intake [for specific methodologies, see Mahaffey and Rice (1997); Mahaffey et al. (2004)]. If we found statistically significant differences in amount of fish consumed per meal from the 24-hr dietary recall by either coastal status (participants who lived in a county that bordered the Pacific or Atlantic Oceans, the Gulf of Mexico, or the Great Lakes vs. those who did not) or data release (1999–2000–2001–2002, or 2003–2004), we calculated separate averages. We generated statistically representative estimates from these data using the statistical weights provided by NCHS and following the relevant analytical guidelines published by NCHS (CDC 2006a).

### Methodology for defining coastal and non-coastal areas

Fish consumption is generally believed to be a major contributor to BHg concentration. We hypothesized that patterns of fish and shellfish consumption would vary between U.S. residents who live on or near the coast (within ~ 25–50 miles) and those who live inland. We further hypothesized that fish consumption patterns, and thus BHg concentrations, may also vary by specific coast (e.g., residents near the Atlantic Coast may have different BHg concentrations than those on the coast of the Gulf of Mexico) and specific inland region (e.g., West vs. Midwest). To test these hypotheses, we categorized NHANES respondents as living in either a coastal or a noncoastal county and further categorized them by eight regions: Atlantic Coast, Northeast, Great Lakes, Midwest, South, Gulf of Mexico, West, and Pacific Coast.

The geographic unit used by NHANES is a county or county equivalent (CDC 2006a); therefore, we limited our definitions of coastal and noncoastal to follow county boundaries. We defined all counties that bordered the Pacific or Atlantic Oceans, the Gulf of Mexico, or any of the Great Lakes as coastal. Additionally, we defined counties that bordered estuaries and bays as coastal, as well as counties whose center point was within approximately 25 miles of any coast even if not directly bordering a coast. [For the list of counties, see Supplemental Material, Table 13 (http://www.ehponline.org/members/2008/11674/suppl.pdf).] We then defined the four coastal regions based on nearest body of water; for example, counties in California, Oregon, Washington, Alaska, and Hawaii that we defined as coastal were categorized as Pacific Coast. We separated noncoastal counties into four inland regions using the U.S. Census regions; for example, noncoastal counties in California, Oregon, Washington, and Alaska along with the entire states of Idaho, Montana, Wyoming, Colorado, New Mexico, Arizona, Utah, and Nevada became the West region (we classified all of Hawaii as coastal). We also designated the entire state of Florida as coastal, split between the Atlantic Coast and the Gulf of Mexico. We designated Miami-Dade County as Atlantic Coast, and Monroe County as Gulf of Mexico. These subdivisions run the risk of small sample sizes; however, the definition of coastal was sufficiently broad to avoid single primary sampling units.

## Results

**Figure 1:**
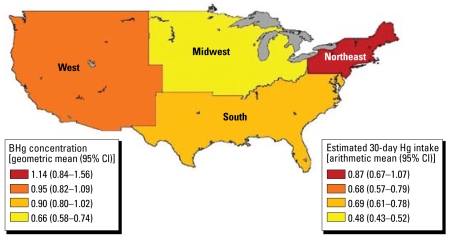
BHg concentration [geometric mean (95% CI) (μg/L)] and estimated 30-day dietary Hg intake [arithmetic mean (95% CI) (μg/kg_bw_)] by U.S. Census region. CI, confidence interval.

**Figure 2:**
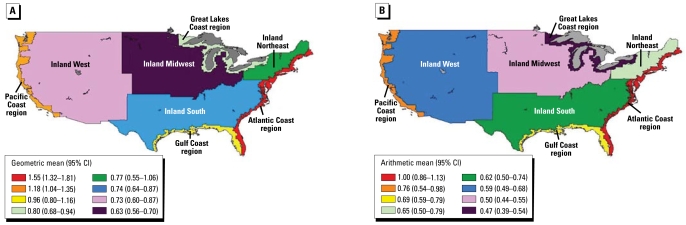
BHg concentration [geometric mean (95% CI) (μg/L)] (*A*) and estimated 30-day Hg intake [arithmetic mean (95% CI) (μg Hg/kg_bw_)] (*B*) by coastal/inland regions. CI, confidence interval.

**Figure 3:**
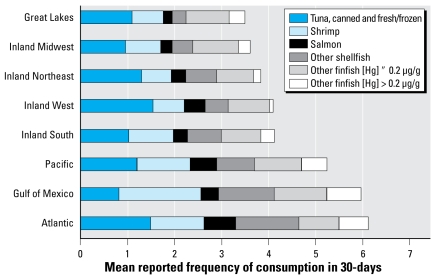
Species and frequency of meals consumed by geographic residence.

**Figure 4:**
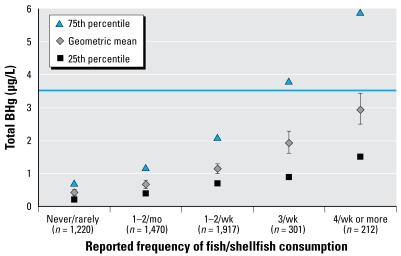
BHg concentration (μg/L) by estimated consumption frequency of fish and shellfish. Blue line identifies adult women's concentration associated with cord BHg ≥ 5.8 μg/L.

Table 1 shows the distributions of estimates of the number of women with BHg concentrations > 3.5 μg/L and > 5.8 μg/L, by region. Analyses indicate that between 1999 and 2004, the Northeast had the highest percentage of women with BHg concentrations above the 3.5 μg/L level of concern (> 19%), whereas the South had the largest estimated number of women (1.21 million) with ≥ 3.5 μg/L BHg because of elevated population in that region. Geometric means (Figure 1) show similar trends, with the highest BHg concentrations in the Northeast, followed by the West, South, and Midwest census regions. In the Northeast, the highest 5% of BHg concentrations exceeded 8.2 μg/L. [For full distributions, see Supplemental Material, Table 2 (http://www.ehponline.org/members/2008/11674/suppl.pdf).] When we included coastal regions in this analysis, additional spatial heterogeneity in BHg (Figure 2A) and estimated 30-day Hg intake (Figure 2B) was apparent, with elevated exposures in all coastal areas relative to their neighboring inland regions except in the Great Lakes. In the coastal areas, the highest 5% of BHg concentrations exceeded 7.2 μg/L, with the Atlantic Coast exceeding 10.9 μg/L. [For the full distributions, see Supplemental Material, Table 4 (http://www.ehponline.org/members/2008/11674/suppl.pdf).] Fish species eaten by survey participants varied by region (Figure 3), with respondents in coastal regions reporting higher frequency of consumption of fish containing higher levels of Hg. BHg concentrations were strongly associated with the frequency of fish consumption (Figure 4). BHg increased with monthly estimated consumption of fish and shellfish over the range of never/rarely to 4 or more times per week. In multiple regression modeling, women from the Atlantic (*p* < 0.01), Pacific (*p* < 0.0001), and Gulf (*p* < 0.0001) coasts had higher BHg concentrations compared with women from the inland West, whereas women from the inland Northeast and inland Midwest had significantly lower BHg levels (*p* < 0.0001). [For the full regression results, see Supplemental Material, Table 11 (http://www.ehponline.org/members/2008/11674/suppl.pdf).]

**Figure 5:**
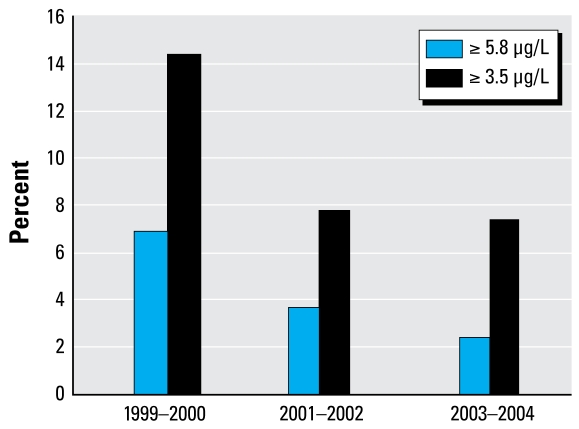
Percentage of women 16–49 years of age having BHg concentrations greater than those associated with exposures considered higher than the U.S. EPA’s RfD for MeHg.

**Figure 6:**
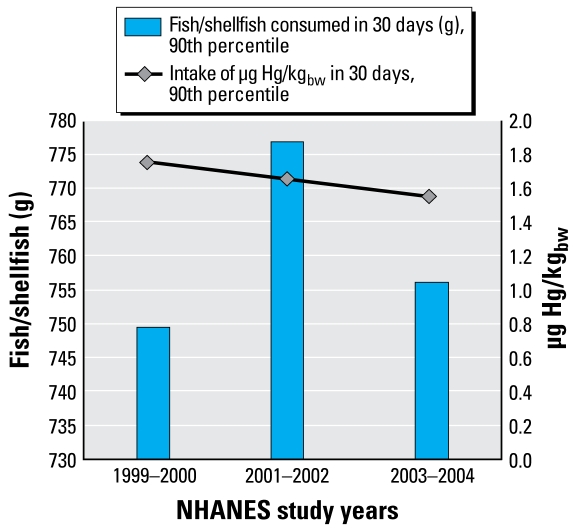
Ninetieth percentiles of estimated 30-day consumption of fish and shellfish (g) and estimated 30-day intake of Hg (μg Hg/kg_bw_) by NHANES study year.

Analysis of temporal trends through simple regression modeling showed no statistically significant difference among the three sets of study years (1999–2000–2001–2002, and 2003–2004) for BHg (*p* = 0.07), estimated 30-day Hg intake (*p* = 0.11), or reported frequency of seafood consumption (*p* = 0.69). However, in multiple regression modeling, adjusting for covariates including coastal/non-coastal residence, the years 1999–2000 had significantly higher BHg levels (*p* < 0.0001) compared with 2003–2004, and 2001–2002 had significantly lower BHg levels (*p* < 0.01) [Supplemental Material, Table 11 (http://www.ehponline.org/members/2008/11674/suppl.pdf)]. Although the analyses did not support the conclusion that there was a general downward trend in BHg concentrations over the 6-year study period, there was a decline in the upper percentiles reflecting the most highly exposed women with BHg concentrations greater than established levels of concern [Supplemental Material, Table 9 (http://www.ehponline.org/members/2008/11674/suppl.pdf)]. In addition, the percentage of examinees with BHg values ≥ 3.5 μg/L and ≥ 5.8 μg/L was much greater in 1999–2000 compared to 2001–2002 and 2003–2004 (Figure 5). We found no consistent trend in fish consumption across the study years. We observed a decrease in the 90th percentile of 30-day estimated intake of Hg through seafood consumption across the study years even though there was no similar decrease in the 90th percentile of 30-day estimated consumption of grams of fish and shellfish (Figure 6). This suggests a shift in consumption to seafood containing less Hg. We did not observe a similar pattern at the mean, suggesting that this shift in seafood consumption occurred mainly with the highest fish and shellfish consumers [Supplemental Material, Table 9 (http://www.ehponline.org/members/2008/11674/suppl.pdf)]. The RfD for Hg intake is 0.1 μg Hg/kg body weight (μg/kg_bw_) per day, or 3.0 μg Hg/kg_bw_ per month (30 days).

Results also showed that self-selected ethnic identity was associated with total BHg concentrations, estimated 30-day Hg intake, and frequency of either finfish or shellfish consumption. For example, BHg levels, reported frequency of seafood consumption, and 30-day Hg intakes were highest among women who designated themselves as being in the “other” category (mostly people whose ancestry is Asian, Native American, Pacific Islands, and the Caribbean Islands). [See also Supplemental Material, Tables 5–7 (http://www.ehponline.org/members/2008/11674/suppl.pdf).] Table 2 presents the percentages of women by race/ethnicity that had ≥ 3.5 and ≥ 5.8 μg/L BHg.

We identified statistically significant relationships between higher income and, respectively, increasing BHg concentration (*p* < 0.0001), estimated 30-day intake of Hg (*p* = 0.008), and 30-day frequency of finfish and shellfish consumption (*p* < 0.0001) through bivariate regressions. [For the distributions of blood total Hg, estimated Hg intake, and frequency of finfish and shellfish consumption by annual income, see Supplemental Material, Tables 6–8 (http://www.ehponline.org/members/2008/11674/suppl.pdf).] In addition, women from families reporting incomes of ≥ $75,000 (the reference category) had statistically higher BHg levels than did women from families with incomes of ≤ $55,000 (*p* < 0.01). In all cases, BHg concentrations were also significantly associated with age and estimated 30-day Hg intake (*p* < 0.0001). Table 3 presents the percentage of women by annual income with ≥ 3.5 and ≥ 5.8 μg/L BHg.

In multiple regression modeling, after adjusting for other factors related to BHg, both race/ethnicity and income remained statistically significant predictors of BHg levels observed in this study [Supplemental Material, Table 11 (http://www.ehponline.org/members/2008/11674/suppl.pdf)]. Non-Hispanic blacks (*p* < 0.0001) and women grouped in the “other” racial category (*p* = 0.002) had significantly higher BHg concentrations than did non-Hispanic whites.

## Discussion

### Regional and coastal variation in BHg concentrations and in fish consumption

Comparisons of the distribution of BHg data with reference values aimed at protecting the fetal nervous system have been made using national-level data (Mahaffey et al. 2004). NHANES data are often used to make population estimates through application of weighting factors to variables of interest such as BHg. Population estimates for U.S. Census regions and their distribution of BHg concentrations indicate that women living in the Northeast had BHg concentrations exceeding levels of concern more often than did women living in the South and West. The lowest Hg exposures were reported among women living in the Midwest.

Because NHANES was not designed to provide population estimates for coastal and noncoastal areas, unbiased estimates for the number of women having BHg concentrations ≥ 3.5 μg/L and ≥ 5.8 μg/L cannot be developed comparing coastal- and noncoastal-residing women. Although the following are not population estimates, they are statistics for a geographic region: Women living in coastal areas were at greater risk of having BHg concentrations ≥ 3.5 μg/L (16.25% for coastal and 5.99% for noncoastal residents) and ≥ 5.8 μg/L (8.11% for coastal and 2.06% for noncoastal residents). Women living near the coastal areas had approximately three to four times greater risk of exceeding acceptable levels of Hg exposure than did noncoastal-dwelling women. There may be some bias in these results due to the weighting issues (see “Materials and Methods”); however, we do not believe that this bias is a major factor underlying these great differences.

MeHg exposures exceeding health-based standards, including U.S. EPA’s RfD (Rice et al. 2003), occurred more commonly among women living in coastal areas. These health-based standards were based on avoiding MeHg-associated delays and deficits in neurologic development of children after *in utero* exposure to MeHg (Mergler et al. 2007; Rice et al. 2003). At higher exposures to MeHg, including the highest concentrations reported during these survey years, the women themselves may risk adverse neuropsychological and neurobehavioral outcomes (Mergler et al. 2007).

Within the United States, people living in coastal areas consume more fish and shellfish than do those living in noncoastal areas and consume fish with higher Hg concentrations. Reports from New York City (McKelvey et al. 2007) and Florida (Denger et al. 1994; Karouna-Renier et al. 2008) support our identification of higher Hg exposures in U.S. coastal areas. This is part of a worldwide pattern. An overall pattern of higher BHg levels has also been reported among people living on U.S. islands [Hawaii (Sato et al. 2006)] and territories [e.g., Puerto Rico (Ortiz-Roque and López-Rivera 2004)]. A similar pattern has been repeated in other islands [Bermuda (Dewailly and Pereg 2004; see also Bermuda Biological Stations for Research 2004), Fiji (Kumar et al. 2006), Seychelles (Myers et al. 2007), and Tahiti (Chateau-Degat 2005; Dewailly et al. 2008)] compared with inland populations. Among these island populations, BHg concentrations at the upper end of the distribution fall into the range of 50 μg/L (~ 250 nmol/L) and higher (Chateau-Degat 2005). In Bermuda, cord BHg concentrations as high as 160 nmol/L (~ 35 μg/L) have been reported (arithmetic mean, 41.3 ± 4.7 nmol/L or 8.0 ± 1.0 μg/L) (Dewailly and Pereg 2004; see also Bermuda Biological Stations for Research 2004).

Higher BHg concentrations in the U.S. Northeast found in this study reflect, in part, more frequent fish and shellfish consumption. Additional variability may be a function of differences in Hg concentrations among species and geographic regions (Sunderland 2007). For example, recent information on “hot spots” for Hg in wildlife tissues (Evers et al. 2007) could be associated with higher Hg concentrations for locally obtained fish. One limitation of the present analysis was the use of a fish-species–specific mean Hg concentration (i.e., nondistributional values) to estimate individual exposure. Although most fish consumed by the U.S. population is not locally obtained (i.e., commercially obtained from diverse regions and countries) (Sunderland 2007), analytical results showing geographic differences in the distribution of BHg could reflect higher Hg concentrations in locally obtained fish within the Northeast states.

### Ethnic group variation on fish intake and BHg concentrations

Ethnic origins were associated with Hg exposures with those designated as “other” (i.e., Asian, Pacific and Caribbean Islander, Native American, Alaska Native, multiracial, and unknown race) having higher BHg concentrations. From additional studies, people of Asian descent whose food choices are influenced by Asian dietary patterns (Kudo et al. 2000; Sechena et al. 2003) tended to consume fish more frequently, in greater variety, and in greater quantity than did non-Asians. The ethnic diversity of the U.S. population is well known. As of 1997, 61% of the Asian population living in the United States was foreign-born (Council of Economic Advisors 1999). By comparison with overall U.S. data, higher BHg concentrations among Asians and islanders were reported for Taiwan (Hsu et al. 2007), Cambodia (Agusa et al. 2007), Fiji (Kumar et al. 2006), and Tahiti (Dewailly et al. 2008).

Within the United States, fish and shellfish consumption, predicting Hg exposure described previously, varies widely, in part a reflection of ethnicity. For example, Asian countries [e.g., Cambodia (Agusa et al. 2007), Taiwan (Hsu et al. 2007; Soong et al. 1991), Japan (Murata et al. 2007; Sakamoto et al. 2007)], island nations [e.g., Bermuda (Dewailly and Pereg 2004; see also Bermuda Biological Stations for Research 2004), Seychelles (Myers et al. 2007), Tahiti (Chateau-Degat 2005), Taiwan (Hsu et al. 2007; Soong et al. 1991), Japan (Murata et al. 2007; Sakamoto et al. 2007)], and some European countries [e.g., Spain (Falcó et al. 2006; Herreros et al. 2008) and the Faroe Islands (Weihe et al. 1996)] have reported fish/shellfish consumption levels greater than average worldwide consumption (World Health Organization 2008).

### Income differences in association with fish intake and BHg concentrations

In contrast to some other environmental exposures [e.g., higher blood lead concentrations] (Mahaffey et al. 1982), BHg concentrations increased with income. This is consistent with other studies in which women from higher income groups were at greater risk of MeHg exposure, as were women living in urban areas (Hightower and Moore 2003; Saint-Phard and Van Dorsten 2006).

### Interactions between income and ethnic group

A more complex association between income and racial/ethnic group may also exist. According to the 1990 U.S. Census (U.S. Census Bureau 2008), the median family income of Japanese-American families exceeded that of non-Hispanic white families. By contrast, the income of Cambodian-American families was lower than that of black families. We could not address whether there is an interaction between belonging to the category designated as “other” and higher income within the NHANES data on BHg levels available at this time, because of sample size limitations.

### Time trends in Hg exposure absent changes in total fish consumption

Our analysis of 30-day Hg intake indicated that there was no consistent trend in fish consumption by women of childbearing age over the 6-year period between 1999 and 2004. Our evaluation of NHANES fish intake data indicated no differences in the mean frequency or amount of particular fish and shellfish species consumed. However, the estimated 30-day Hg intake decreased at the 90th percentile and higher, whereas total fish consumption did not, which suggests a shift in fish species consumed. The BHg data indicated a reduction of the higher end of the distribution of BHg between the first 2-year interval (the 1999 and 2000 examinees) compared with the subsequent 4-year interval (the 2001–2004 examinees).

The basis for these differences could possibly reflect spillover from the federal fish advisory program (U.S. EPA 2008b) in terms of total fish and shellfish consumption. A recent analysis of a nationally representative study specifically addressing fish-consumption patterns did not support this suggestion (Bradbury 2007).

The four fish species listed in the federal advisory [swordfish, shark, tilefish, and king mackerel (U.S. EPA 2008b)] were rarely reported by the 5,465 women in this analysis. It is clear that these four fish species contributed little to Hg exposure in this general population of U.S. women. Individual states with higher Hg exposures [e.g., Hawaii (Sato et al., 2006), Florida (Karouna-Renier et al. 2008)] and greater fish consumption [Florida (Denger et al. 1994)] have substantially broader fish consumption advisories (e.g., Hawaii and Florida) aimed at reducing Hg exposure from high-Hg–containing species obtained locally (Florida Department of Health 2007; U.S. EPA 2008b). Despite the federal advisory’s emphasis on four species of highly contaminated fish (U.S. EPA 2008b) and the states’ emphasis on game-fish, the most commonly consumed finfish in the United States is tuna. Interpretation of Hg exposure from tuna was complicated by the specific wording of the dietary questions asked of the NHANES examinees, which did not differentiate between light or skipjack tuna and albacore tuna. The latter contains approximately three times more Hg than does the former: 0.38 μg/g for frozen and fresh tuna, 0.35 μg/g for canned albacore, and 0.12 μg/g for canned light tuna (Mahaffey et al. 2008).

### Changes in MeHg exposure over time

During the past decade, the U.S. EPA initiated substantial interventions aimed to reduce Hg releases and exposures (U.S. EPA 2008a) and issued advisories to limit consumption of high-Hg fish (U.S. EPA 2008b). Because of worldwide atmospheric distribution and subsequent deposition of Hg, local conditions and locally caught fish are not the main contributors to Hg intake for most people (Sunderland 2007). Although there are economic indications that consumption of some species of fish may have decreased in response to these advisories (Shimshack et al. 2007), Hg exposures may not follow a similar time trend despite regulatory efforts to reduce Hg exposures. A recent analysis of a nationally representative study specifically addressing fish-consumption patterns did not support this suggestion (Bradbury 2007). Our analysis of NHANES data calculating 30-day Hg intake indicated that there was no consistent trend in fish consumption by women of childbearing age over the 6-year period between 1999 and 2004.

## Conclusions

Significant geographic differences in BHg concentrations occurred within the United States: We found highest exposures in coastal areas and the Northeast census region. In the Northeast, 19% of women had BHg concentrations ≥ 3.5 μg/L. The highest 5% of BHg concentrations exceeded 8.2 μg/L in the Northeast and 7.2 μg/L in coastal areas, concentrations more than twice the 3.5 μg/L level of concern. BHg levels were predicted by the quantity and type of fish consumed. Over the 6-year period (1999–2004), the frequency of elevated BHg levels among women of childbearing age declined without a significant change in quantities of fish and shellfish consumed. This pattern suggests a more discerning series of choices in type of fish eaten rather than an overall reduction in fish consumption. Within all geographic regions, women at highest risk of elevated Hg exposures were more affluent and more likely to be of Asian or island ethnicity.

## Figures and Tables

**Table 1:** Percentages of examinees and population estimates (in millions) of women with BHg concentrations ≥ 3.5 μg/L and ≥ 5.8 μg/L, by U.S. Census region and coastal status.

	U.S. Census region					Coastal status^a^	
BHg	Nation	Northeast	South	Midwest	West	Coastal	Noncoastal
Percent ≥ 3.5 μg/L (SE)	10.4 (1.0)	19.3 (4.1)	10.8 (1.0)	2.8 (0.9)	10.3 (1.3)	16.3 (1.8)	6.0 (1.0)
No. of women ≥ 3.5 μg/L (millions)	6.92	2.15	2.85	0.41	1.51		
Percent ≥ 5.8 μg/L (SE)	4.7 (0.7)	9.0 (2.3)	4.6 (1.0)	1.2 (0.6)	4.9 (0.9)	8.1 (1.2)	2.1 (0.4)
No. of women ≥ 5.8 μg/L (millions)	3.1	1.0	1.21	0.17	0.72		

a NHANES was not designed to provide population estimates for coastal and noncoastal areas; therefore unbiased estimates of the number of women having BHg concentrations ≥ 3.5 μg/L and to ≥ 5.8 μg/L cannot be developed.

**Table 2:** Percentages of examinees and population estimates (in millions) of women with BHg concentrations ≥ 3.5 μg/L and ≥ 5.8 μg/L by race/ethnicity.

BHg	All	Mexican American	Other Hispanic	Non-Hispanic white	Non-Hispanic black	Other race^a^
Percent ≥ 3.5 μg/L (SE)	10.4 (1.0)	4.9 (0.7)	9.4 (2.7)	10.0 (1.4)	10.3 (1.7)	27.4 (3.1)
No. of women ≥ 3.5 μg/L (millions)	6.92	0.30	0.44	4.4	0.90	0.91
Percent ≥ 5.8 μg/L (SE)	4.7 (0.7)	1.4 (0.3)	2.9 (1.4)	4.6 (0.9)	4.1 (1.1)	15.7 (2.9)
No. of women ≥ 5.8 μg/L (millions)	3.1	0.08	0.14	2.0	0.36	0.52

a Includes people whose ancestry is Asian, Native American, Pacific Islands, and the Caribbean Islands.

**Table 3:** Percentages of examinees and population estimates (in millions) of women with BHg concentrations ≥ 3.5 μg/L and ≥ 5.8 μg/L by annual income.

BHg	All	$0–$9,999	$10,000–$19,999	$20,000–$34,999	$35,000–$54,999	$55,000–$74,999	≥ $75,000
Percent ≥ 3.5 μg/L (SE)	10.4 (1.0)	4.3 (1.3)	5.4 (1.4)	6.8 (1.4)	9.6 (1.6)	10.5 (1.7)	16.2 (2.2)
No. of women ≥ 3.5 μg/L (millions)	6.92	0.18	0.40	0.80	1.11	0.92	2.72
Percent ≥ 5.8 μg/L (SE)	4.7 (0.7)	1.0 (0.5)	1.7 (0.7)	2.9 (0.8)	5.3 (1.3)	6.0 (1.4)	7.1 (1.3)
No. of women ≥ 5.8 μg/L (millions)	3.1	0.04	0.12	0.34	0.62	0.53	1.20

## References

[b1-ehp-117-47] Agency for Toxic Substances and Disease Research (1999). ATSDR Announces Updated Toxicological Profile for Mercury.

[b2-ehp-117-47] Agusa T, Kunito T, Iwata H, Monirith I, Chamnan C, Tana TS (2007). Mercury in hair and blood from residents of Phnom Penh (Cambodia) and possible effect on serum hormone levels. Chemosphere.

[b3-ehp-117-47] Bellanger TM, Caesar EM, Trachtman L (2000). Blood mercury levels and fish consumption Louisiana. J La State Med Soc.

[b4-ehp-117-47] Bermuda Biological Stations for Research (2004). Mobile Laboratory in Bermuda: Final Report.

[b5-ehp-117-47] Bradbury S (2007). Awareness of Mercury among Pregnant Women (U.S. FDA National Study). Proceedings of the 2007 National Forum on Contaminants in Fish.

[b6-ehp-117-47] Butler Walker J, Houseman J, Seddon L, McMullen E, Tofflemire K, Mills C (2004). Maternal and umbilical cord blood levels of mercury, lead, cadmium, and essential trace elements in Arctic Canada. Environ Res.

[b7-ehp-117-47] CDC (Centers for Disease Control and Prevention) (2006a). Analytical Guidelines. National Health and Nutrition Examination Survey Data.

[b8-ehp-117-47] CDC (Centers for Disease Control and Prevention) (2006b). Data and Documentation. National Health and Nutrition Examination Survey Data.

[b9-ehp-117-47] CDC (Centers for Disease Control and Prevention) (2006c). Research Data Center Guidelines. National Health and Nutrition Examination Survey Data.

[b10-ehp-117-47] Chateau-Degat M-L (2005). Neurological signs of ciguatera disease: evidence of their persistency. Portrait Épidémiologique de la Ciguatera dans le Pacifique-sud [PhD Thesis].

[b11-ehp-117-47] Council of Economic Advisors (1999). Changing America. Indicators of Social and Economic Well-Being by Race and Hispanic Origin.

[b12-ehp-117-47] Denger R, Adams C, Moss S, Mack S (1994). Per Capita Fish and Shellfish Consumption in Florida. Florida Agriculture and Market Research Center.

[b13-ehp-117-47] Dewailly É, Chateau-Degat L, Subas E (2008). Fish consumption and health in French Polynesia. Asia Pac J Clin Nutr.

[b14-ehp-117-47] Dewailly É, Pereg D (2004). The Atlantis Mobile Laboratory in Bermuda.

[b15-ehp-117-47] European Union (2002). Risks to Health and the Environment Related to the Use of Mercury Products. Report by Risk and Policy Analysts.

[b16-ehp-117-47] Evers DC, Han YJ, Driscol CT, Hamman NC, Goodale MW, Lambert KF (2007). Biological mercury hotspots in the northeastern United States and southwestern Canada. BioScience.

[b17-ehp-117-47] Falcó G, Llobet JN, Bocio A, Domingo JL (2006). Daily intake of arsenic, cadmium, mercury, and lead by consumption of edible marine species. J Agric Food Chem.

[b18-ehp-117-47] Florida Department of Health (2007). Florida Fish Consumption Advisories.

[b19-ehp-117-47] Herreros MA, In̆igo-Nun̆ez, Sanchez-Perez E, Encinas T, Gozales-Bulnes A (2008). Contribution of fish consumption to heavy metals exposure in women of childbearing age from a Mediterranean country (Spain). Food Chem Toxicol.

[b20-ehp-117-47] Hightower JM, Moore D (2003). Mercury levels in high-end consumers of fish. Environ Health Perspect.

[b21-ehp-117-47] Hsu CS, Liu PL, Chien LC, Chou SY, Han BC (2007). Mercury concentration and fish consumption in Taiwanese pregnant women. BJOG.

[b22-ehp-117-47] Joint Food and Agriculture Organization/World Health Organization Expert Committee on Food Additives (2003). Summary of Evaluations Performed by the Joint FAO/WHO Expert Committee on Food Additives: Methylmercury.

[b23-ehp-117-47] Karouna-Renier N, Rao KR, Lanzai JL, Rivers SD, Wilson PA, Hodges DK (2008). Mercury levels and fish consumption practices in women of child-bearing age in the Florida Panhandle. Environ Res.

[b24-ehp-117-47] Knobeloch L, Anderson H, Imma P, Petersa D, Smith A (2005). Fish consumption advisory awareness, and hair mercury levels among women of childbearing age. Environ Res.

[b25-ehp-117-47] Kudo Y, Falcigila GA, Couch SC (2000). Evolution of meal patterns and food choices of Japanese-American females born in the United States. Eur J Clin Nutr.

[b26-ehp-117-47] Kumar M, Aalbersberg B, Mosley L (2006). Mercury Levels in Fijian Seafoods and Potential Health Implications. Report for World Health Organization.

[b27-ehp-117-47] Mahaffey KR (2004). Fish and shellfish as dietary sources of methylmercury and the omega-3 fatty acids. Eicosahexaenoic acid and docosahexaenoic acid: risks and benefits. Environ Res.

[b28-ehp-117-47] Mahaffey KR, Annest JL, Roberts J, Murphy RS (1982). National estimates of blood lead levels: United States, 1976–1980: association with selected demographic and socioeconomic factors. N Engl J Med.

[b29-ehp-117-47] Mahaffey KR, Clickner RP, Bodurow CC (2004). Blood organic mercury and dietary mercury intake: National Health and Nutrition Examination Survey, 1999 and 2000. Environ Health Perspect.

[b30-ehp-117-47] Mahaffey KR, Clickner RP, Jeffries RA (2008). Methylmercury and omega-3 fatty acids: co-occurrence of dietary sources with emphasis on fish and shellfish. Environ Res.

[b31-ehp-117-47] Mahaffey KR, Rice G (1997). Calculation of mercury concentrations in fish dishes. Mercury Study Report to Congress. An Assessment of Exposure to Mercury in the United States.

[b32-ehp-117-47] McDowell M, Dillon CF, Osterloh J, Bolger PM, Pelizzari E, Fernando R (2005). Hair mercury levels in U.S. children and women of childbearing age: reference range data from NHANES 1999–2000. Environ Health Perspect.

[b33-ehp-117-47] McKelvey W, Gwynn RC, Jeffery N, Kass D, Thorpe LE, Garg RK (2007). A biomonitoring study of lead, cadmium, and mercury in the blood of New York city adults. Environ Health Perspect.

[b34-ehp-117-47] Mergler D, Anderson HA, Chan LH, Mahaffey KR, Murray M, Sakamoto M (2007). The Panel on Health Risks and Toxicological Effects of Methylmercury. Methylmercury exposure and health effects in humans: a worldwide concern. Ambio.

[b35-ehp-117-47] Morrissette J, Takser L, St-Amour G, Smargiassi A, Lafond J, Mergler D (2004). Temporal variation of blood and hair mercury levels in pregnancy in relation to fish consumption history in a population living along the St. Lawrence River. Environ Res.

[b36-ehp-117-47] Murata K, Dakeishi M, Shimada M, Satoh H (2007). Assessment of intrauterine methylmercury exposure affecting child development: messages from the newborn. Tohoku J Exp Med.

[b37-ehp-117-47] Myers GJ, Davison PW, Strain JJ (2007). Nutrient and methyl mercury exposure from consuming fish. J Nutr.

[b38-ehp-117-47] NRC (National Research Council) (2000). Toxicity of Methylmercury. Committee on Toxicological Effects of Methylmercury, Board on Environmental Studies and Toxicology, Commission on Life Sciences.

[b39-ehp-117-47] NRC (National Research Council) (2006). Selections to Balance Benefits and Risks. Seafood Choices. Committee on Nutrient Relationships in Seafood, Food and Nutrition Board, Institute of Medicine.

[b40-ehp-117-47] Ortiz-Roque C, López-Rivera Y (2004). Mercury contamination in reproductive age women in a Caribbean island: Vieques. J Epidemiol Community Health.

[b41-ehp-117-47] Rice DC, Schoeny R, Mahaffey KR (2003). Methods and rationale for derivation of a reference dose for methylmercury by the U.S. EPA. Risk Anal.

[b42-ehp-117-47] Saint-Phard D, Van Dorsten B (2006). Mercury toxicity: clinical presentations in musculoskeletal medicine. Orthopedics.

[b43-ehp-117-47] Sakamoto M, Kaneoka T, Murata K, Nakai K, Satoh H, Akagi H (2007). Correlations between mercury concentrations in umbilical cord tissue and other biomarkers of fetal exposure to methylmercury in the Japanese population. Environ Res.

[b44-ehp-117-47] Sato RL, Li GG, Shaha S (2006). Antepartum seafood consumption and mercury levels in newborn cord blood. Am J Obstet Gynecol.

[b45-ehp-117-47] Sechena R, Liao S, Lorenzana R, Nakano C, Polissar N, Fenske R (2003). Asian American and Pacific Islander seafood consumption—a community-based study in King County, Washington. J Expo Anal Environ Epidemiol.

[b46-ehp-117-47] Shimshack JP, Ward MB, Beaty TKM (2007). Mercury advisories: information, education, and fish consumption. J Environ Econ Manag.

[b47-ehp-117-47] Soong YK, Tseng R, Liu C, Lin PW (1991). Lead, cadmium, arsenic, and mercury levels in maternal and fetal cord blood. J Formos Med Assoc.

[b48-ehp-117-47] Stern AH, Gochfeld M, Weisel C, Burger J (2001). Mercury and methylmercury exposure in the New Jersey pregnant population. Arch Environ Health.

[b49-ehp-117-47] Stern AH, Smith AE (2003). An assessment of the cord blood: maternal blood methylmercury ratio: implications for risk assessment. Environ Health Perspect.

[b50-ehp-117-47] Sunderland E (2007). Mercury exposure from domestic and imported estuarine and marine fish in the U.S. seafood market. Environ Health Perspect.

[b51-ehp-117-47] U.S. Census Bureau (2008). Census 1990.

[b52-ehp-117-47] U.S. EPA (U.S. Environmental Protection Agency) (2001). Integrated Risk Information System.

[b53-ehp-117-47] U.S. EPA (U.S. Environmental Protection Agency) (2008a). Mercury.

[b54-ehp-117-47] U.S. EPA (U.S. Environmental Protection Agency) (2008b). What You Need to Know about Mercury in Fish and Shellfish.

[b55-ehp-117-47] Weihe P, Grandjean P, Debes F, White R (1996). Health implications for Faroe Islanders of heavy metals and PCBs from pilot whales. Sci Total Environ.

[b56-ehp-117-47] World Health Organization (2008). Global and Regional Food Consumption Patterns and Trends: Availability and Consumption of Fish.

